# Who benefits from orthogeriatric treatment? Results from the Trondheim hip-fracture trial

**DOI:** 10.1186/s12877-016-0218-1

**Published:** 2016-02-19

**Authors:** Anders Prestmo, Ingvild Saltvedt, Jorunn L. Helbostad, Kristin Taraldsen, Pernille Thingstad, Stian Lydersen, Olav Sletvold

**Affiliations:** Department of Neuroscience, Norwegian University of Science and Technology (NTNU), Trondheim, Norway; Department of Geriatrics, St Olav Hospital, University Hospital of Trondheim, Trondheim, Norway; Clinic of Clinical Services, St Olav Hospital, University Hospital of Trondheim, Trondheim, Norway; Regional Centre for Child and Youth Mental Health and Child Welfare, Norwegian University of Science and Technology (NTNU), Trondheim, Norway

**Keywords:** Geriatric assessment, Hip fracture, Orthogeriatric, Sub group

## Abstract

**Background:**

Hip fracture patients are heterogenous. Certain patient characteristics are associated with poorer prognosis, but less is known about differences in response to treatment among subgroups. The Trondheim Hip Fracture trial found beneficial effects on mobility and function from comprehensive geriatric care (CGC) compared to traditional orthopaedic care (OC). The aim of this study was to explore differences in response to CGC among subgroups in this trial.

**Methods:**

Secondary analysis of the complete dataset from Trondheim Hip Fracture Trial, a randomised controlled trial including 397 home-dwelling older adults (≥70 years) with a hip fracture. Subgroups were age (over/under 80 years), gender, fracture type (intra-/extracapsular), and pre-fracture instrumental ADL (i-ADL) (defined as over/under 45 on the Nottingham Extended ADL scale). Dependent variables were mobility (Short Physical Performance Battery), personal ADL (p-ADL) (Barthel Index), i-ADL (Nottingham Extended ADL scale), cognition (Mini-Mental Status Examination), four and 12 months after hip fracture. Data were analysed by linear mixed models with interactions (treatment, time, and subgroup), reporting treatment effects being clinically and statistically significant within and between subgroups.

**Results:**

Analyses within subgroups showed beneficial effects of CGC on mobility and i-ADL either at four or twelve months in all subgroups except for males, extra-capsular fractures and patients with impaired pre-fracture i-ADL. Beneficial effect on p- ADL was found in patients < 80 years, intra-capsular fractures and patients with impaired pre-fracture i-ADL. Effects on cognition were found in patients < 80 years and men.

The interaction analyses showed that CGC had statistically significant better treatment effect on i-ADL for younger participants at four months (*p* = 0.004), on p-ADL both at four (*p* = 0.037) and twelve months (*p* = 0.045) and mobility at twelve months (*p* = 0.021), for participants with intracapsular as compared to extracapsular fractures, and on i-ADL at twelve months for participants with higher pre-fracture function (*p* = 0.012).

**Conclusion:**

Contrary to our hypothesis that the most vulnerable patients would benefit the most from CGC, we found the intervention effect was most pronounced in younger, female participants with higher pre-fracture i-ADL function.

**Trial rigistration:**

ClinicalTrials.gov registration number: NCT00667914.

**Electronic supplementary material:**

The online version of this article (doi:10.1186/s12877-016-0218-1) contains supplementary material, which is available to authorized users.

## Background

Hip fractures are common with more than 1.3 million fractures annually world wide [1]. A hip fracture represents a major burden both for the individual patient and the society [2], consequences are often reduced survival, impaired function and problems with independent living. Older age is associated with not regaining basic mobility following a hip fracture. After a hip fracture mortality is higher among men. However, there are conflicting results on gender differences in the regaining of function [3, 4]. Several studies have reported that risk of reduced mobility after a fracture is higher in patients with low pre-fracture mobility and in those with extra-capsular fractures [5, 6].

Geriatric patients and hip fracture patients share features such as high age, comorbidities, functional limitations, and frailty [7]. Therefore, orthogeriatric treatment models where geriatricians and orthopaedic surgeons collaborate have been developed. As summarised in literature reviews, orthogeriatric treatment models have shown reduction of delirium, post-surgery complication rates and mortality [8, 9], and improved mobility [10]. A recent paper from The Trondheim Hip Fracture Trial reported that treatment of home-dwelling hip-fracture patients with comprehensive geriatric care (CGC) throughout the entire hospital stay gave statistically significant and clinically meaningful better mobility, personal activities of daily living (p-ADL), instrumental ADL (i-ADL), and cognition, and was also cost-effective as compared to traditional care [11].

Previous studies indicate beneficial effects of comprehensive geriatric care (CGC) for hip-fracture patients in general [11, 12], but less is known about benefits of CGC in targeted subgroups. Although a number of prognostic factors for functional outcomes are relatively well known, we have not found any randomised controlled trial (RCT) evaluating treatment effects of CGC versus traditional orthopaedic care (OC) related to subgroup characteristics.

When planning for The Trondheim Hip-Fracture Trial, we hypothesised that benefits of a comprehensive and individualised orthogeriatric treatment programme were independent of age, gender and fracture type, and that focusing on functional recovery in the CGC group would especially benefit those with more severe pre-fracture impairments.

The aim of the present study is to explore post hoc if treatment effects of CGC as compared to OC depend on subgroups defined by age, gender, type of fracture or pre-fracture function. This will be studied separately for the outcome measures of mobility, p-ADL, i-ADL, and cognition.

## Methods

### Trial design and patients

**Fig. 1:**
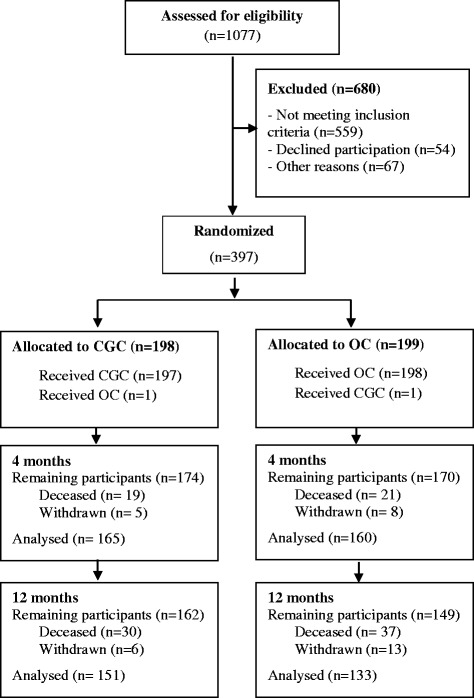
Patient flow chart. CGC = Comprehensive Geriatric Care; OC = Orthopedic Care

The Trondheim Hip-fracture Trial is a prospective RCT recruited patients at St. Olav University Hospital in Trondheim, Norway between April 2008 and December 2010, last follow-up assessment was completed January 2012. The protocol, the intervention and clinical outcomes from the study have been published previously [11, 13–15]. Home-dwelling patients 70 years or older who had been able to walk 10 m prior to the hip-fracture were eligible. Patients with pathological fractures, multiple trauma, short life expectancy, living permanently in a nursing home, or already participating in the study were excluded. A nurse in the emergency room screened the patients for eligibility, collected informed written consent by either the patients or their next of kin, and randomised the patients. A web-based computer-generated service prepared by the Norwegian University of Science and Technology (NTNU) was used that randomised patients in a ratio of 1:1 and blocks of unknown size was used. Patients were randomised to receive CGC or OC and were transferred to the allocated wards directly after randomisation. Blinding of patients and staff was not possible, while assessors were partly blinded during follow-up [11]. Patient flow is shown in Fig. 1.

### Treatment

As described previously [14] patients in both groups received the same perioperative treatment. In most patients surgery was performed in spinal anaesthesia. Arthroplasty was used for dislocated intracapsular fractures (Garden type 3 or 4) while Garden type 1 or 2 fractures were mainly treated with a two-screw fixation. A sliding hip screw system was used for extracapsular fractures except for some sub trochanteric fractures that were treated with intramedullary nailing. Most patients were allowed full weight-bearing postoperatively, except for 17 (9 %) and 20 (10 %) in the CGC and OC groups respectively, who got restrictions. Most of these had sub-trochanteric fractures or other fractures that were considered to be unstable according to the fixation method and reposition, or the surgeon had judged the bone as too osteoporotic for weight-bearing even after fixation.

In the orthopaedic trauma ward OC patients received treatment according to national and international guidelines [16–18]. In the geriatric ward patients were treated pre- and postoperatively using CGC performed as a multidimensional interdisciplinary diagnostic process focusing on the patients’ medical, mental, social and functional situation. The CGC emphasised medical assessment including review of drug regimen, pain relief, hydration, nutrition, elimination, and assessment of fall risk and osteoporosis. In addition the interdisciplinary team focused on early mobilisation and rehabilitation and early individualised discharge planning.

The primary health care services were responsible for follow-up after discharge from hospital in both groups. Neither group was routinely offered hospital-based follow-up, except for selected patients who were offered follow-up in the orthopaedic outpatient clinic as decided by the orthopaedic surgeons. We developed an integrated plan for treatment and follow-up for each patient [14]. 

### Measurements

P-ADL and i-ADL before the fracture and at four and 12 months were assessed by the Barthel Index (BI; 0 to 20 points; 20 best score) and the Nottingham Extended ADL Scale (NEAS; 0–66 points; 66 best score) [19, 20]. The median pre-fracture NEAS score was 45, and patients with NEAS scores ≥45 before the fracture were regarded well-functioning, while those with scores < 45 were regarded impaired in i-ADL. Data were obtained by interviewing the patient or, if he/she was not able to respond, their next of kin. Mobility at four and 12 months was assessed by the Short Physical Performance Battery (SPPB; 0 to 12 points; 12 best score) [21]. Cognition was assessed by the Mini Mental Status Examination (MMSE; 0–30 points; 30 best score) [22]. The American Society of Anaesthesiologists (ASA) score [23] was used as preoperative risk score. Medical information was collected from hospital records.

### Statistical analysis

Sample size was calculated for the analysis of the main effect (without subgroups). For an estimated effect size of 1.0 point in mean SPPB score at four months after surgery, and with an α level of 0.05 304 patients were needed for 80 % power. To allow for an estimated 20 % drop-out rate 380 patients were required, in the end a total of 397 were included. Sample size estimations were not carried out for the post hoc analyses. There were no planned or unplanned formal interim analyses. An independent clinical trials unit reviewed emerging safety data (mortality and serious adverse events), and the assumptions underlying the sample size calculation when 200 patients had been recruited [11].

The statistical analysis plan that was completed before performing any data analysis, also involved subgroup analyses regarding pre-fracture function. Subgroup analyses on age, gender and fracture type were decided posthoc.

Differences between subgroups were analysed by linear mixed models with interactions between treatment, time, and subgroup, using SPPB, BI, NEAS, and MMSE as dependent variables. Independent variables were time, group allocation (CGC vs OC), and age (70 to 79 vs ≥80 years), gender, fracture type (intra- vs extra-capsular) and pre-fracture function (median NEAS < 45 vs ≥45). An interaction between the subgroup and the treatment effect implies a three-way interaction (between time, treatment and subgroup). The magnitude of the three-way interaction is not of practical interest, but the interest lies in the effect of treatment group at four and 12 months. Hence, at each time point, we report the treatment effect within subgroups, and the difference in treatment effect between subgroups.

The results within and between subgroups are presented as mean scores for differences with 95 % Confidence Intervals (CI). Differences in treatment effect between subgroups are reported with CIs and p-values for the relevant two-way interactions. Two-sided *p*-values <0.05 were considered statistically significant. For evaluation of whether test score differences are clinically meaningful previously reported reference values were used: SPPB ≥ 0.5 points [24], BI ≥ 1.4 points [25], NEAS ≥ 2.4 points [26], and MMSE ≥ 2 points [27].

Analyses were performed using SPSS 21.

### Ethics

The study was approved by the Regional Committee of Ethics in Medical Research (REK4.2008.335), the Norwegian Social Science Data Services (NSD19109) and the Norwegian Directorate of Health (08/5814). ClinicalTrials.Gov registry number was NCT00667914.

## Results

### Baseline characteristics

As shown in Fig. 1 a total of 1077 patients were screened for eligibility, of these 559 did not meet inclusion criteria, 54 declined participation and 67 were not included from other reasons, while 397 were randomised 198 to CGC and 199 to OC.

The two groups were comparable regarding baseline characteristics (Table 1). Mean age was 83 years, three of four patients were female, and 60 % were living alone. More than 50 % in both groups had an ASA score of 3 or higher. About 60 % had intra-capsular fractures, of whom 76 (63.9 %) in the CGC and 89 (69.3 %) in the OC were operated with arthroplasty (Table 1) (*p* = 0.37). Baseline characteristics for each subgroup has been added as Additional file 1.

**Table 1:** Baseline characteristics

	Geriatric		Orthopaedic	
	*n* = 198		*n* = 199	
Age (years) - mean (SD)	83.4 (5.4)		83.2 (6.4)	
Sex (female) - *n* (%)	145 (73.2)		148 (74.4)	
Sheltered housing - *n* (%)	26 (13.5)		20 (10.3)	
Living alone - *n* (%)	115 (58.1)		124 (62.3)	
Barthel Index (0–20) - mean (SD)	18.3 (2.3)		18.1 (2.8)	
NEAS (0–66) - mean (SD)	42.5 (17.7)		41.9 (17.5)	
ASA score (1–5) mean (SD)	2.5 (0.7)		2.6 (0.7)	
ASA score				
1 or 2 (healthy or mild systemic disease) - *n* (%)	89 (45.0)		82 (41.2)	
3 (severe systemic disease) - *n* (%)	103 (52.0)		106 (53.3)	
4 or 5 (severe systemic disease or moribund) - *n* (%)	6 (3.0)		11 (5.5)	
Previous diagnoses				
Heart disease - *n* (%)	97 (49.0)		89 (44.7)	
Stroke - *n* (%)	49 (24.7)		57 (28.6)	
Diabetes - *n* (%)	23 (11.6)		28 (14.1)	
Dementia - *n* (%)	27 (13.6)		26 (13.1)	
Cancer - *n* (%)	53 (26.8)		43 (21.6)	
Kidney disease - *n* (%)	18 (9.1)		9 (4.5)	
Fracture type				
Femoral neck - *n* (%)	119	(60.1)	127	(63.8)
Extra capsular fracture- *n* (%)	79	(39.9)	72	(36.1)
Surgery				
Hemi prosthesis - *n* (%)	76	(38.4)	88	(44.2)
Bone plates and -screws - *n* (%)	69	(34.8)	63	(31.7)
Screws – *n* (%)	38	(19.2)	32	(16.1)
Other^a^- *n* (%)	15	(7.6)	16	(8.0)

*ASA* American Society of Anaesthesiologists

^a^Including patients treated with combinations of surgery or no surgery at all due to death

### Clinically meaningful treatment effects of CGC versus OC within subgroups

**Fig. 2:**
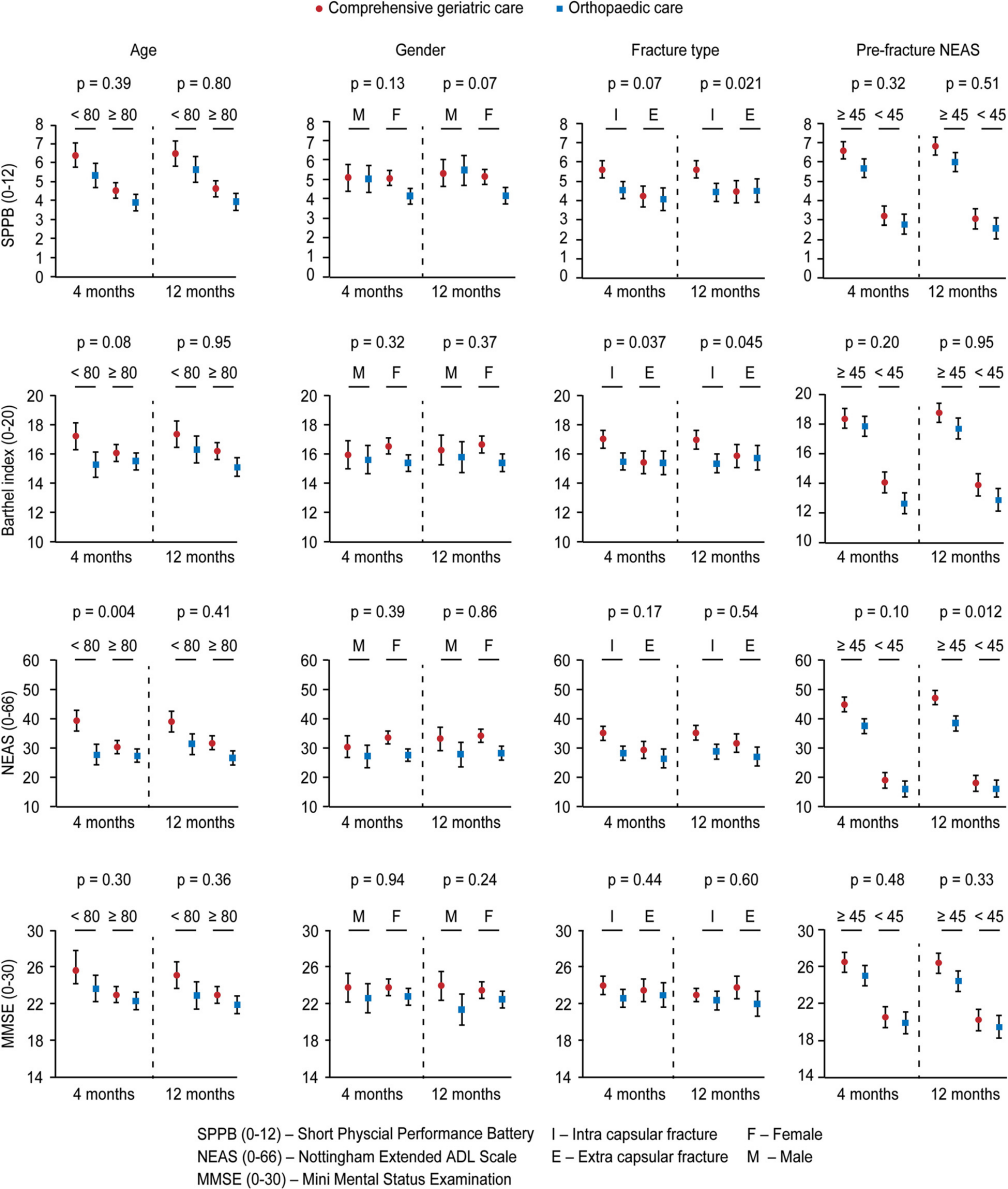
Results of mixed model analysis: Estimated outcome in each subgroup and treatment, with 95 % confidence interval. P-values are reported for interaction between subgroup and treatment. For example, *p* = 0.004 for age and NEAS at 4 months: The treatment effect (difference in NEAS) for patients aged < 80 is significantly larger than the difference for patients aged ≥ 80

Table 2 and Fig. 2 show that at four months patients aged 70–79 years treated with CGC had better performance on SPPB, BI and NEAS than patients treated with OC, and at 12 months better performance on NEAS and MMSE. CGC patients ≥80 years of age had better SPPB scores at four and 12 months, and better NEAS score at 12 months than the OC patients.

**Table 2:** Results of linear mixed model analysis

	CGC	OC			CGC	OC			2-way interaction	
	(mean)	(mean)	GD (95 % CI)	*p*-value	(mean)	(mean)	GD (95 % CI)	*p*-value	GD (95 % CI)	*p*-value
					4 months (*n* = 325)					
		Age 70–79 (*n* = 98)			Age 80 or older (*n* = 227)					
SPPB	6.41	5.32	1.09 (0.19–1.99)	0.017	4.53	3.91	0.62 (0.03–1.20)	0.039	0.47 (−0.60 to 1.55)	0.39
Barthel	17.19	15.27	1.92 (0.66–3.18)	0.003	16.05	15.49	0.56 (−0.26–1.37)	0.18	1.36 (−0.14 to 2.86)	0.08
NEAS	39.18	27.73	11.44 (6.54–16.35)	<0.0001	30.30	27.36	2.94 (−0.25 to 6.13)	0.07	8.50 (2.65–14.35)	0.004
MMSE	25.64	23.66	1.97 (−0.06–4.02)	0.06	23.02	22.33	0.70 (−0.62–2.02)	0.30	1.28 (−1.14–3.71)	0.30
		Male (*n* = 78)			Female (*n* = 247)					
SPPB	5.09	5.03	0.06 (−0.93–1.05)	0.91	5.07	4.14	0.94 (0.37–1.50)	0.001	0.88 (−0.26–2.02)	0.13
Barthel	15.94	15.60	0.34 (−1.03–1.72)	0.63	16.51	15.36	1.15 (0.36–1.94)	0.005	0.81 (−0.78–2.40)	0.32
NEAS	30.43	27.13	3.30 (−2.08–8.68)	0.23	33.60	27.58	6.02 (2.93–9.12)	0.0001	2.72 (−3.48–8.93)	0.39
MMSE	23.75	22.64	1.11 (−1.13–3.34)	0.33	23.79	22.78	1.01 (−0.27–2.29)	0.12	0.09 (−2.48–2.67)	0.94
		Intra-capsular fracture (*n* = 203)			Extra-Capsular fracture (*n* = 122)					
SPPB	5.61	4.54	1.07 (0.46–1.69)	0.001	4.21	4.07	0.14 (−0.66–0.94)	0.73	0.93 (−0.08–1.95)	0.07
Barthel	17.00	15.47	1.53 (0.66–2.39)	0.001	15.40	15.38	0.02 (−1.10–1.14)	0.97	1.51 (0.09–2.92)	0.037
NEAS	35.02	28.16	6.85 (3.48–10.23)	0.0001	29.35	26.40	2.94 (−1.45–7.34)	0.19	3.91 (−1.64–9.45)	0.17
MMSE	24.01	22.62	1.39 (−0.01–2.80)	0.05	23.47	22.97	0.50 (−1.30–2.30)	0.59	0.90 (−1.39–3.19)	0.44
		Pre-fracture NEAS ≥ 45 (*n* = 178)			Pre-fracture NEAS < 45 (*n* = 147)					
SPPB	6.59	5.66	0.93 (0.28–1.59)	0.005	3.22	2.78	0.45 (−0.26–1.15)	0.22	0.49 (0.48–1.45)	0.32
Barthel	18.36	17.83	0.53 (−0.40–1.47)	0.27	14.06	12.64	1.42 (0.42–2.42)	0.005	0.89 (−0.48–2.26)	0.20
NEAS	44.77	37.44	7.34 (3.83–10.84)	<0.0001	19.06	16.03	3.03 (−0.73–6.78)	0.11	4.31 (−0.82–9.45)	0.10
MMSE	26.44	25.01	1.43 (−0.08–2.94)	0.06	20.51	19.88	0.62 (−1.01–2.25)	0.45	0.80 (−1.42–3.03)	0.48
					12 months (n = 284)					
		Age 70–79 (*n* = 87)			Age 80 or older (*n* = 197)					
SPPB	6.48	5.64	0.84 (−0.09–1.78)	0.08	4.63	3.93	0.70 (0.08–1.31)	0.027	0.14 (−0.98–1.26)	0.80
Barthel	17.34	16.29	1.05 (−0.24–2.35)	0.11	16.20	15.09	1.11 (0.26–1.95)	0.011	0.05 (−1.49–1.60)	0.95
NEAS	39.01	31.33	7.68 (2.62–12.64)	0.003	31.75	26.63	5.12 (1.79–8.45)	0.003	2.56 (−3.50–8.62)	0.41
MMSE	25.13	22.90	2.23 (0.16–4.30)	0.035	23.00	21.92	1.07 (−0.27–2.42)	0.12	1.16 (−1.31–3.63)	0.36
		Male (*n* = 65)			Female (*n* = 219)					
SPPB	5.33	5.47	−0.14 (−1.20–0.91)	0.79	5.13	4.17	0.96 (0.37–1.55)	0.001	1.10 (−0.11–2.31)	0.07
Barthel	16.25	15.78	0.47 (−0.99–1.93)	0.53	16.62	15.38	1.24 (0.42–2.06)	0.003	0.77 (−0.90–2.44)	0.37
NEAS	33.07	27.78	5.29 (−0.47–11.04)	0.07	34.10	28.21	5.89 (2.69–9.08)	0.0003	0.60 (−5.98–7.19)	0.86
MMSE	23.95	21.36	2.59 (0.30–4.87)	0.027	23.50	22.49	1.01 (−0.29–2.31)	0.13	1.58 (−1.05–4.01)	0.24
		Intra-capsular fracture (*n* = 177)			Extra-Capsular fracture (*n* = 107)					
SPPB	5.61	4.42	1.19 (0.54–1.84)	0.0003	4.45	4.51	−0.06 (−0.090–0.78)	0.89	1.25 (0.19–2.31)	0.021
Barthel	16.95	15.33	1.62 (0.72–2.52)	0.0004	15.85	15.72	0.13 (−1.02–1.27)	0.83	1.49 (0.04–2.95)	0.045
NEAS	35.15	28.76	6.40 (2.86–9.83)	0.0004	31.66	27.05	4.61 (0.11–9.11)	0.045	1.79 (−3.94–7.51)	0.54
MMSE	22.94	22.37	1.14 (−0.29–2.58)	0.12	23.77	22.00	1.77 (−0.06–3.61)	0.06	0.63 (−1.70–2.96)	0.60
		Pre-fracture NEAS ≥ 45 (*n* = 164)			Pre-fracture NEAS < 45 (*n* = 120)					
SPPB	6.81	5.98	0.83 (0.16–1.51)	0.016	3.06	2.57	0.49 (−0.26–1.24)	0.20	0.34 (−0.67–1.35)	0.51
Barthel	18.74	17.68	1.05 (0.10–2.01)	0.031	13.89	12.88	1.01 (−0.04–2.06)	0.06	0.05 (−1.37–1.47)	0.95
NEAS	47.11	38.38	8.73 (5.14–12.32)	<0.0001	18.08	16.18	1.90 (−2.06–5.85)	0.35	6.83 (1.48–12.17)	0.012
MMSE	26.33	24.42	1.91 (0.38–3.44)	0.015	20.24	19.47	0.77 (−0.91–2.45)	0.37	1.14 (−1.14–3.41)	0.33

The two-way interaction term GD is the difference in effect of CGC vs OC between the subgroups, for example 1.09–0.62 = 0.47 for SPPB, age70-79 vs age 80+, 4 months

*CGC* Comprehensive Geriatric Care, *OC* orthopaedic care, *GD* Group difference, *SPPB* Short Physical Performance Battery, *Barthel* Barthel Index, *NEAS* Nottingham Extended ADL Scale, *MMSE* Mini Mental Status Examination

In females there were clinically meaningful treatment effects in favour of CGC for SPPB and NEAS at four and 12 months. For men the MMSE scores for the CGC group was better at 12 months, but there were no other statistically significant differences at four or 12 months.

At four and 12 months patients with intra-capsular fractures treated with CGC had better scores on SPPB, BI and NEAS. Patients with extra-capsular fractures had a better treatment effect of CGC than OC only for NEAS at 12 months.

CGC patients with pre-fracture NEAS ≥45 had better scores on SPPB and NEAS at four and 12 months and better MMSE scores at 12 months. Among patients with pre-fracture NEAS <45 there was better scores for BI at four months and no statistically significant differences between the CGC and OC group at 12 months.

### Clinically meaningful treatment effects of CGC versus OC between subgroups

The analysis showed that CGC was better than OC for patients 70–79 years of age as compared to patients ≥80 years for NEAS at four months, while there were no differences at 12 months. In patients with intra-capsular as compared to extra-capsular fractures, CGC was better for BI at four and 12 months and for SPPB at 12 months. In patients with pre-fracture NEAS ≥45 as compared to NEAS < 45, CGC was better for NEAS at 12 months (Table 2).

## Discussion

We have previously reported that treating home-dwelling hip-fracture patients in an orthogeriatric ward improves mobility, p-ADL, i-ADL and cognition more than treating patients in an orthopaedic ward. Our overall aim of the present study was to explore treatment effects on functional measures between subgroups of the hip-fracture population. This post hoc study have shown that home-dwelling hip-fracture patients irrespective of age, gender, type of fracture or pre-fracture function have better effect of CGC than OC in one or more functional outcomes, and that these group differences are of clinical importance. Nevertheless, the results demonstrated only minor differences in functional outcomes between the CGC and OC group among men, patients with extracapsular fractures, and those with impaired i-ADL before the fracture. The interaction analyses showed that CGC had statistically significant better treatment effect on i-ADL for younger participants at four months (*p* = 0.004), on p-ADL both at four (*p* = 0.037) and twelve months (*p* = 0.045) and mobility at twelve months (*p* = 0.021), for participants with intracapsular fractures as compared to extracapsular fractures, and on i-ADL at twelve months for participants with higher pre-fracture function (*p* = 0.012).

We have not found other publications studying if effects of orthogeriatric care differ in subgroups of patients. However, our overall results indicating somewhat better effects of CGC than OC irrespective of subgroup are in line with a Cochrane review on comparison of comprehensive geriatric assessment with general medical care in hospitalised acutely sick elderly patients, that showed that the benefits were related to treatment in a geriatric ward per se and not a consequence of admission criteria like age and other factors [28].

Previous studies have shown that older patients have poorer functional recovery than younger patients after hip-fractures [29]. In the present study there were statistically and/or clinically meaningful differences between the CGC and OC groups independent of age group. For patients ≥ 80 years the effect of CGC was more pronounced at 12 months. The between-subgroup analysis showed a significant better effect of CGC on i-ADL at four months in patients 70–79 years as compared to patients ≥80. This difference between age groups disappears after one year where the superior effect of CGC is fairly similar regardless of age. The change is mainly due to improved i-ADL in the older group by CGC, but not in OC. Our interpretation is that patients ≥80 need more time to improve, and that the effect of CGC may persist beyond discharge due to a better definition of treatment goals, better discharge planning or a better individual plan for rehabilitation.

Arinzon & al [30] have previously found that both men and women improve mobility during hip-fracture rehabilitation, while other studies have found better prognosis for female hip-fracture patients [31]. In our study we found that while female CGC patients had statistically and clinically significant better mobility and i-ADL at four and 12 months, the only effect for male CGC patients was on better cognition at 12 months. This is in line with the findings in our main publication from the study where we found improved cognition at 12 months in the CGC group [11]. All outcomes in the present study measure different aspects of function. Cognitive impairment is frequently shown among sick, frail elderly patients and often interrelated with general health status [32]. Nevertheless, we have no plausible explanation for the gender difference found in the subgroup analyses. Still, there was no significant effect of gender in the between-group analyses, possibly due to lack of statistical power. Further research particularly designed to assess gender effects of rehabilitation is warranted in order to improve treatment outcomes particularly in male hip-fracture patients.

Better effect in favour of CGC on the intra-capsular fracture group was found at both four and 12 months, while for the extra-capsular fracture group there was only a rather small effect on i-ADL at four and 12 months. The interaction analyses confirmed these findings by revealing increased benefit of CGC versus OC on several outcomes for intra-capsular as compared to extra-capsular fractures. Our findings also support previous studies showing that in general prognosis is poorer for extra-capsular as compared to intra-capsular fractures [33]. One explanation may be that these patients have a larger trauma with more soft tissue damage and needing more extensive surgery. Further research is needed in order to improve the outcome for this patient group.

When planning the study we hypothesised that patients with high pre-fracture i-ADL scores would benefit least from CGC, and the group with impairments in i-ADL before the fracture would benefit the most. We found however a marginal effect on p-ADL at four months and no effect on other outcomes for the group with low pre-fracture i-ADL scores, while CGC improved mobility and i-ADL at four and 12 months for the group with high pre-fracture i-ADL scores. Thus, our hypothesis was not supported. The interaction analysis showed that CGC was significantly better for patients with high pre-fracture i-ADL scores than for those having low pre-fracture i-ADL scores on i-ADLduring follow-up. This is in line with findings from the The Oslo Orthogeriatric Trial that also showed beneficial effect on mobility among those being fittest before the fracture [34]. The median NEAS score was only 45 at baseline, indicating that most participants actually had a functional decline before the fracture. Thus, our findings support previous studies reporting that impaired pre-fracture function appears to be a consistent predictor of unfavourable outcomes and not regaining mobility in older persons with hip-fractures. [5, 6, 29, 33, 35].

The strengths of the study are the randomised controlled design and large sample size, and that we had a plan for analysis of subgroup effects based on pre-fracture function before the study started. The main weakness is the post-hoc design with choice of the other subgroups based upon literature review (defined after the main outcomes of the study were known). We have found no consensus on how to categorise patients with impaired function before the fracture. As there was a ceiling effect of BI before the fracture with a median score of 20 while the median NEAS score was 45 of 66 points, we selected pre-fracture NEAS to categorise pre-fracture function. In lack of established cut-off values for the NEAS we dichotomised by the median of the baseline score. Other weaknesses are lack of power for some subgroups, and that a large number of analyses have been performed increasing the chance of Type I error. However, according to the viewpoints of Rothman, we have not adjusted for multiple testing [36, 37]. The outcome measures in this study were chosen to represent aspects of function, while subjective reported outcomes as for example quality of life were not studied. Thus, conclusions should not be generalised to other domains. The study was exploratory, and further studies primarily designed to study effect of treatment on subgroups have to be undertaken to confirm findings.

## Conclusion

Our main results were that in home-dwelling hip-fracture patients all subgroups of patients benefit of CGC on one or more functions (mobility, i- and p-ADL, and cognition), irrespective of age, gender, type of fracture and pre-fracture function. These findings support the implementation of CGC for different subgroups of home-dwelling hip-fracture patients. Contrary to our hypothesis that the most vulnerable patients would benefit the most from CGC, we found the intervention effect was most pronounced in younger, female participants with higher pre-fracture i-ADL function. Our results also show that there is need of further research, especially on extra-capsular fractures, on males, and patients with functional decline before the fracture.

### Availability of supporting data

Data files are not available due to participants’ confidentiality.

## Additional file

Additional file 1:
**Baseline tables for the different subgroups.** (DOC 142 kb)

## Abbreviations

ADL: activities of daily living
BI: barthel index
CGC: comprehensive geriatric care
I-ADL: instrumental ADL
MMSE: mini mental status and examination
NEAS: Nottingham extended ADL scale
OC: standard orthopaedic care
p-ADL: personal ADL
SPPB: short physical performance battery
