# Supplementary material for: Who benefits from orthogeriatric treatment? Results from the Trondheim hip-fracture trial
Source: BMC Geriatr. 2016 Feb 19;16:49. doi: 10.1186/s12877-016-0218-1 (PMC4761133; doi:10.1186/s12877-016-0218-1)
Supplement: Additional file 1: — Baseline tables for the different subgroups. (DOC 142 kb) [file 12877_2016_218_MOESM1_ESM.doc]

**BASELINE CHARACTERISTICS FOR EACH SUBGROUP STUDIED**

**Table 1 A: Baseline characteristics - Age**

|  |  |  | **<80 years**  **n= 114** | | | | **80 years or older**  **n= 293** | | | |
| --- | --- | --- | --- | --- | --- | --- | --- | --- | --- | --- |
|  |  |  | **CGC**  **n=53** | | **OC**  **n=61** | | **CGC**  **n=145** | | **OC**  **n=138** | |
| **Age (years) - mean (SD)** | | | 75.8 | (2.7) | 75.5 | (2.9) | 86.2 | (3.8) | 86.6 | (4.1) |
| **Sex (female) - n (%)** | | | 40 | (75.5) | 47 | (77.0) | 105 | (72.4) | 101 | (73.2) |
| **Sheltered housing - n (%)** | | | 6 | (11.8) | 2 | (3.3) | 20 | (14.2) | 18 | (13.5) |
| **Living alone - n (%)** | | | 23 | (43.4) | 27 | (44.3) | 92 | (63.4) | 97 | (74.3) |
| **Barthel Index (0-20) - mean (SD)** | | | 19.1 | (2.0) | 18.2 | (3.2) | 18.0 | (2.4) | 18.4 | (2.3) |
| **NEAS (0-66) - mean (SD)** | | | 49.2 | (15.8) | 43.0 | (19.2) | 40.0 | (17.8) | 41.4 | (16.6) |
| **ASA score (1-5) mean(SD)** | | | 2.4 | (0.6) | 2.3 | (0.7) | 2.6 | (0.7) | 2.7 | (0.7) |
| **ASA score (1-5) – n (%)** | | |  | |  | |  | |  | |
| **1 or 2 – healthy or mild systemic disease** | | | 32 | (60.4) | 34 | (55.8) | 57 | (39.3) | 48 | (34.8) |
| **3- severe systemic disease** | | | 20 | (37.7) | 26 | (42.6) | 83 | (57.2) | 80 | (58.0) |
| **4 or 5 - severe systemic disease or moribund** | | | 1 | (1.9) | 1 | (1.6) | 5 | (3.4) | 10 | (7.2) |
| **Fracture type** | | |  |  |  |  |  |  |  |  |
| **Femoral neck - n (%)** | | | 36 | (67.9) | 43 | (70.5) | 83 | (57.2) | 84 | (60.9) |
| **Trochanteric- n (%)** | | | 13 | (24.5) | 17 | (27.9) | 53 | (36.6) | 41 | (29.7) |
| **Sub trochanteric- n (%)** | | | 4 | (7,5) | 1 | (1.6) | 9 | (6.2) | 13 | (9.4) |
| **Surgery** | | |  |  |  |  |  |  |  |  |
| **Hemiprosthesis - n (%)** | | | 22 | (41.5) | 31 | (50.8) | 54 | (37.2) | 58 | (42.0) |
| **Osteosynthesis- n (%)** | | | 31 | (58.5) | 30 | (49.2) | 89 | (61.4) | 78 | (56.5) |
| **No surgery- n (%)** | | | 0 | (0) | 0 | (0) | 2 | (1.4) | 2 | (1.5) |

ASA- American Society of Anaesthesiologists . NEAS-Nottingham Extended ADL Scale

**Table 1B: Baseline characteristics – fracture type**

|  |  |  | **Intra capsular fractures**  **n=246** | | | | **Extracapsular fractures**  **n= 151** | | | |
| --- | --- | --- | --- | --- | --- | --- | --- | --- | --- | --- |
|  |  |  | **CGC**  **n=119** | | **OC**  **n=127** | | **CGC**  **n=79** | | **OC**  **n=72** | |
| **Age (years) - mean (SD)** | | | 83.2 | (5.8) | 83.1 | (6.7) | 83.7 | (5.8) | 83.3 | (5.7) |
| **Sex (female) - n (%)** | | | 91 | (76.5) | 94 | (74.0) | 54 | (68.4) | 54 | (75.0) |
| **Sheltered housing - n (%)** | | | 13 | (17.6) | 13 | (15.7) | 11 | (11.0) | 9 | (7.3) |
| **Living alone - n (%)** | | | 66 | (55.5) | 81 | (63.8) | 49 | (62.0) | 43 | (59.7) |
| **Barthel Index (0-20) - mean (SD)** | | | 18.6 | (2.1) | 18.2 | (2.9) | 17.9 | (2.6) | 18.5 | (2.2) |
| **NEAS (0-66) - mean (SD)** | | | 42.7 | (17.6) | 41.6 | (17.8) | 42.2 | (18.1) | 42.4 | (17.0) |
| **ASA score (1-5) mean(SD)** | | | 2.4 | (0.7) | 2.6 | (0.8) | 2.7 | (0.6) | 2.5 | (0.6) |
| **ASA score (1-5) – n (%)** | | |  | |  | |  | |  | |
| **1 or 2 – healthy or mild systemic disease** | | | 61 | (51.3) | 50 | (39.4) | 28 | (35.4) | 32 | (44.5) |
| **3- severe systemic disease** | | | 55 | (46.2) | 67 | (52.8) | 48 | (60.8) | 39 | (54.2) |
| **4 or 5 - severe systemic disease or moribund** | | | 3 | (2.5) | 10 | (7.9) | 3 | (3.8) | 1 | (1.4) |
| **Fracture type** | | |  |  |  |  |  |  |  |  |
| **Femoral neck - n (%)** | | | 119 | (100) | 127 | (100) | 0 | (0) | 0 | (0) |
| **Trochanteric- n (%)** | | | 0 | 0 | 0 | 0 | 66 | (83.5) | 58 | (80.6) |
| **Sub trochanteric- n (%)** | | | 0 | 0 | 0 | 0 | 13 | (16.5) | 14 | (19.4) |
| **Surgery** | | |  |  |  |  |  |  |  |  |
| **Hemiprosthesis - n (%** | | | 76 | (63.9) | 88 | (69.3) | 0 | 0 | 1 | (1.4) |
| **Osteosynthesis- n (%)** | | | 42 | (35.3) | 38 | (29.9) | 78 | (98.7) | 70 | (97.2) |
| **No surgery- n (%)** | | | 1 | (0,8) | 1 | (0.8) | 1 | (1.3) | 1 | (2.8) |

ASA- American Society of Anaesthesiologists . NEAS-Nottingham Extended ADL Scale

**Table 1C: Baseline characteristics - gender**

|  |  |  | **Male**  **n=104** | | | | | | | | **Female**  **n= 293** | | | |
| --- | --- | --- | --- | --- | --- | --- | --- | --- | --- | --- | --- | --- | --- | --- |
|  |  |  | **CGC n=53** | | **OC=51** | | | | | | **CGC=145** | | **OC=148** | |
| **Age (years) - mean (SD)** | | | 83.3 | (5.6) | 83.2 | | | | | (6.0) | 83.5 | (5.9) | 83.2 | (6.5) |
| **Sheltered housing - n (%)** | | | 5 | (10.6) | 5 | | | | (10.4) | | 21 | (14.6) | 15 | (10.4) |
| **Living alone - n (%)** | | | 27 | (50.9) | 20 | | | (40.8) | | | 87 | (60.4) | 103 | (69.6) |
| **Barthel Index (0-20) - mean (SD)** | | | 18.3 | (2.3) | 18.0 | | (3.2) | | | | 18.3 | (2.3) | 18.4 | (2.5) |
| **NEAS (0-66) - mean (SD)** | | | 42.2 | (17.4) | 41.1 | | (17.7) | | | | 42.6 | (17.9) | 42.1 | (17.5) |
| **ASA score (1-5) mean(SD)** | | | 2.7 | (0.6) | 2.6 | (0.8 | | | | | 2.4 | (0.7) | 2.6 | (0.7) |
| **ASA score (1-5) – n (%)** | | |  | |  | | | | | |  | |  | |
| **1 or 2 – healthy or mild systemic disease** | | | 18 | (34.0) | 17 | (33.4) | | | | | 71 | (49.0) | 65 | (43.9) |
| **3- severe systemic disease** | | | 32 | (60.4) | 30 | (58.8) | | | | | 71 | (49.0) | 76 | (51.3) |
| **4 or 5 - severe systemic disease or moribund** | | | 3 | (5.7) | 4 | (7.8) | | | | | 3 | (2.1) | 7 | (4.8) |
| **Fracture type** | | |  |  |  |  | | | | |  |  |  |  |
| **Femoral neck - n (%)** | | | 28 | (52.8) | 33 | (64.7) | | | | | 91 | (62.8) | 94 | (63.5) |
| **Trochanteric- n (%)** | | | 22 | (41.5) | 15 | (29.4) | | | | | 44 | (30.3) | 43 | (29.1) |
| **Sub trochanteric- n (%)** | | | 3 | (5.7) | 3 | (5.9) | | | | | 10 | (6.9) | 11 | (7.4) |
| **Surgery** | | |  |  |  |  | | | | |  |  |  |  |
| **Hemiprosthesis - n (%** | | | 16 | (30.2) | 20 | (39.2) | | | | | 60 | (41.4) | 69 | (46.6) |
| **Osteosynthesis- n (%)** | | | 37 | (69.8) | 30 | (58.8) | | | | | 83 | (57.2) | 78 | (52.7) |
| **No surgery- n (%)** | | | 0 | 0 | 1 | (2.0) | | | | | 2 | (1.4) | 1 | (0.7) |

ASA- American Society of Anaesthesiologists . NEAS-Nottingham Extended ADL Scale

**Table 1D: Baseline characteristics - function**

|  |  |  | **Pre-fracture NEAS ≥45**  **n=196** | | | | | | | | **Pre-fracture NEAS < 45**  **n= 201** | | | |
| --- | --- | --- | --- | --- | --- | --- | --- | --- | --- | --- | --- | --- | --- | --- |
|  |  |  | **CGC**  **n=99** | | **OC**  **n=97** | | | | | | **CGC**  **n=99** | | **OC**  **n=102** | |
| **Age (years) - mean (SD)** | | | 81.9 | (5.3) | 82.2 | | | | | (6.5) | 84.9 | (6.0) | 84.2 | (6.1) |
| **Sex (female) - n (%)** | | | 74 | (74.7) | 74 | | | | | (76.3) | 71 | (71.7) | 74 | (72.5) |
| **Sheltered housing - n (%)** | | | 7 | (7.4) | 5 | | | | (5.3) | | 19 | (19.6) | 15 | (15.2) |
| **Living alone - n (%)** | | | 57 | (57.6) | 64 | | | (66.0) | | | 58 | (58.6) | 60 | (58.8) |
| **Barthel Index (0-20) - mean (SD)** | | | 19.8 | (0.5) | 19.6 | | (1.0) | | | | 16.8 | (2.5) | 16.8 | (3.1) |
| **NEAS (0-66) - mean (SD)** | | | 57.9 | (5.7) | 56.5 | | (6.2) | | | | 26.6 | (10.4) | 27.0 | (11.7) |
| **ASA score (1-5) mean(SD)** | | | 2.3 | (0.6) | 2.4 | (0.7) | | | | | 2.7 | (0.7) | 2.7 | (0.7) |
| **ASA score (1-5) – n (%)** | | |  | |  | | | | | |  | |  | |
| **1 or 2 – healthy or mild systemic disease** | | | 61 | (61.6) | 51 | (52.6) | | | | | 28 | (28.3) | 31 | (30.4) |
| **3- severe systemic disease** | | | 38 | (38.4) | 44 | (45.4) | | | | | 65 | (65.7) | 62 | (60.8) |
| **4 or 5 - severe systemic disease or moribund** | | | 0 | (0.0) | 2 | (2.1) | | | | | 6 | (6.1 | 9 | (8.8) |
| **Fracture type** | | |  |  |  |  | | | | |  |  |  |  |
| **Femoral neck - n (%)** | | | 57 | (57.6) | 62 | (63.9) | | | | | 62 | (62.6) | 65 | (63.7) |
| **Trochanteric- n (%)** | | | 35 | (35.3) | 26 | (26.8) | | | | | 31 | (31.3) | 32 | (31.4) |
| **Sub trochanteric- n (%)** | | | 7 | (7.1) | 9 | (9.3) | | | | | 6 | (6.1) | 5 | (4.9) |
| **Surgery** | | |  |  |  |  | | | | |  |  |  |  |
| **Hemiprosthesis - n (%** | | | 34 | (34.3) | 43 | (44.3) | | | | | 42 | (42.4) | 46 | (45.1) |
| **Osteosynthesis- n (%)** | | | 65 | (65.7) | 54 | (55.7) | | | | | 55 | (55.6) | 54 | (52.9) |
| **No surgery- n (%)** | | | 0 | (0) | 0 | (0) | | | | | 2 | (2.0) | 2 | (2.0) |

ASA- American Society of Anaesthesiologists . NEAS-Nottingham Extended ADL Scale
